# Supplementary material for: Susceptibility of Melanoma Cells to Targeted Therapy Correlates with Protection by Blood Neutrophils
Source: Cancers (Basel). 2024 May 2;16(9):1767. doi: 10.3390/cancers16091767 (PMC11083732; doi:10.3390/cancers16091767)
Supplement: Supplementary file 1 [file cancers-16-01767-s001.zip › cancers-2979326-supplementary.pdf]

Supplementary Materials

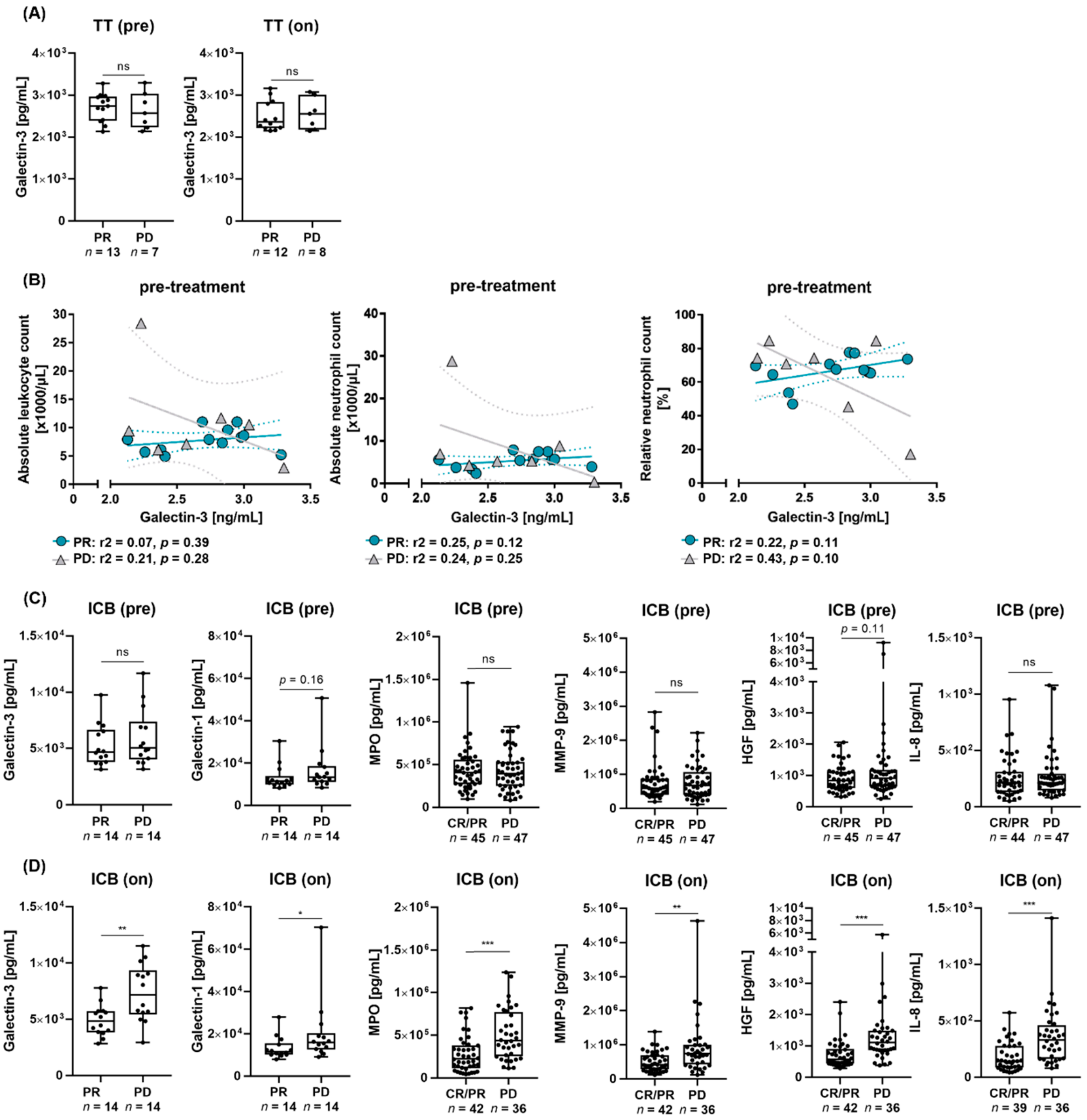

Figure S1. Association of serum markers with clinical outcome in patients with advanced metastatic melanoma receiving targeted therapy or immunotherapy. (**A**) Comparison of serum galectin-3 concentration (pg/mL) of responders (PR) and non-responders (PD) prior (TT (pre)) and during (TT (on)) treatment with first-line targeted therapy. (**B**) Correlation of galectin-3 serum concentration with absolute leukocyte count, absolute neutrophil count and relative neutrophil count pre-treatment comparing PR and PD. Responders display a positive correlation (p = 0.11, r^2 = 0.22) and non-responders a negative correlation (p = 0.10, r^2 = 0.43) of relative neutrophil count with galectin-3 secretion. In total, 50 patients with metastatic melanoma pre-treatment (PR n = 34; PD n = 16) and 32 patients on-treatment (PR n = 16; PD n = 6) were included in the targeted therapy cohort. (**C**), (**D**) Comparison of serum galectin-3, galectin-1, MPO, MMP-9, HGF and IL-8 concentration (pg/mL) of responders (CR and PR) and non-responders (PD) (**C**) prior (ICB (pre)) and (**D**) during (ICB (on)) treatment with first-line immunotherapy. Non-responders are by trend characterized by higher pre-therapeutic galectin-1 (p = 0.16) and HGF (p = 0.11) levels compared to responders. Responders showed significantly lower on-treatment levels of galectin-3, galectin-1, MPO, MMP-9 and IL-8 compared to non-responders. In total, 92 patients with metastatic melanoma (CR n = 5; PR n = 40; PD n = 47) pre-treatment and 78 patients (CR n = 4; PR n = 38; PD n = 36) on-treatment were included in the immunotherapy cohort. ns, $p > 0.05$, * $p \leq 0.05$, ** $p \leq 0.01$ and *** $p \leq 0.001$.

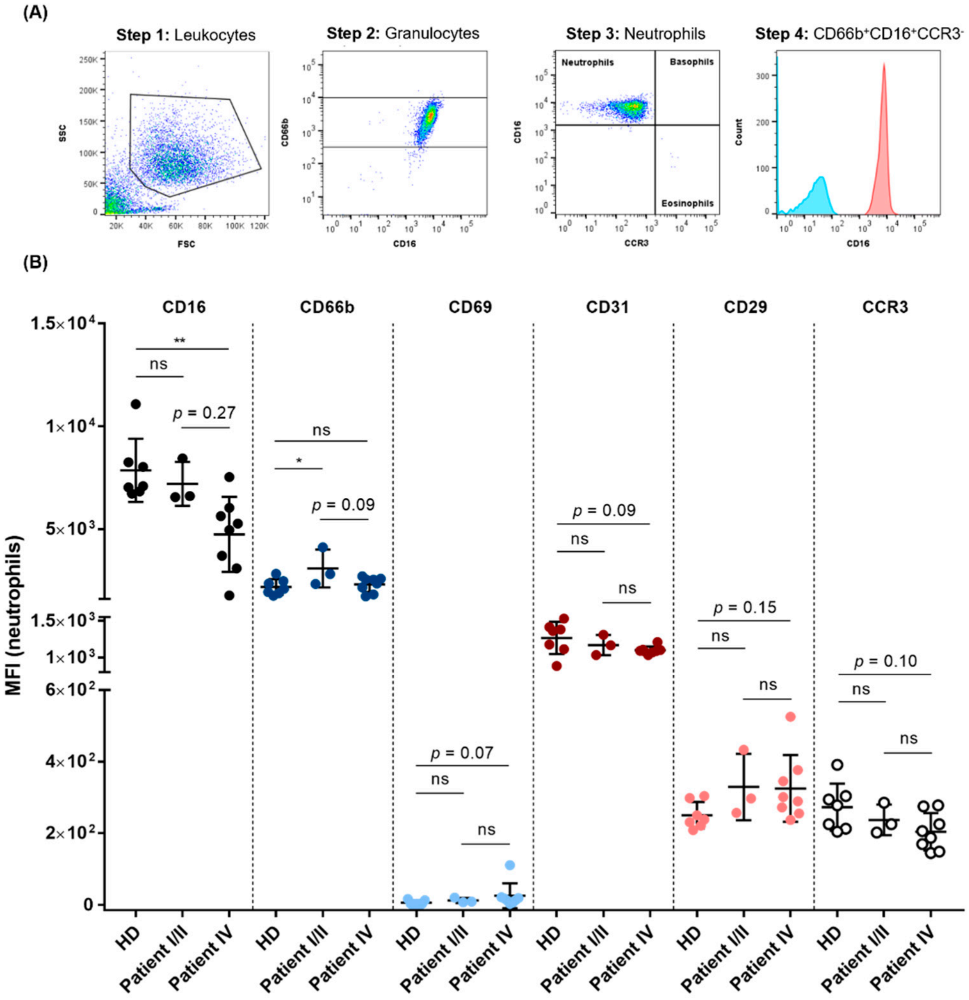

Figure S2. Gating strategies for *ex vivo* neutrophil phenotyping and neutrophil phenotypic characterization. (**A**) Representative dot plots and histogram for the identification of freshly isolated CD66b+CD16+CCR3- neutrophils. Gating strategy was used as described before (Wendlinger et al. [35]). Leukocytes were gated for singlets (not shown), followed by gating on side scatter (SSC) and forward scatter (FSC) (Step 1). Granulocytes were identified as CD66b-positive cells (Step 2). Next, neutrophils were defined as $CD16^+$ $CCR3^-$ cells (Step 3). In the last step, the expression of the target epitope (here: CD16) on neutrophils (red) was displayed in a histogram compared to unstained neutrophils (blue) (Step 4). (**B**) Reduced CD16 expression on neutrophils from stage IV melanoma patients compared to healthy donors. Phenotypic characterization of freshly isolated peripheral blood neutrophils of stage IV and stage I/II melanoma patients compared to healthy donors (HD). Neutrophils from stage IV melanoma patients were analyzed prior therapy initiation. Median fluorescence intensity (MFI) determination for the expression of CD16, CD66b, CD69, CD31, CD29 and CCR3 (CD193) of neutrophils. Each dot represents an individual donor. Neutrophils from stage IV melanoma patients showed reduced CD16 expression compared to healthy donors. The expression analysis included seven HD, three patients with early-stage melanoma (Patient I/II) and eight patients with advanced melanoma (Patient IV). ns, $p > 0.05$, * $p \leq 0.05$ and ** $p \leq 0.01$.

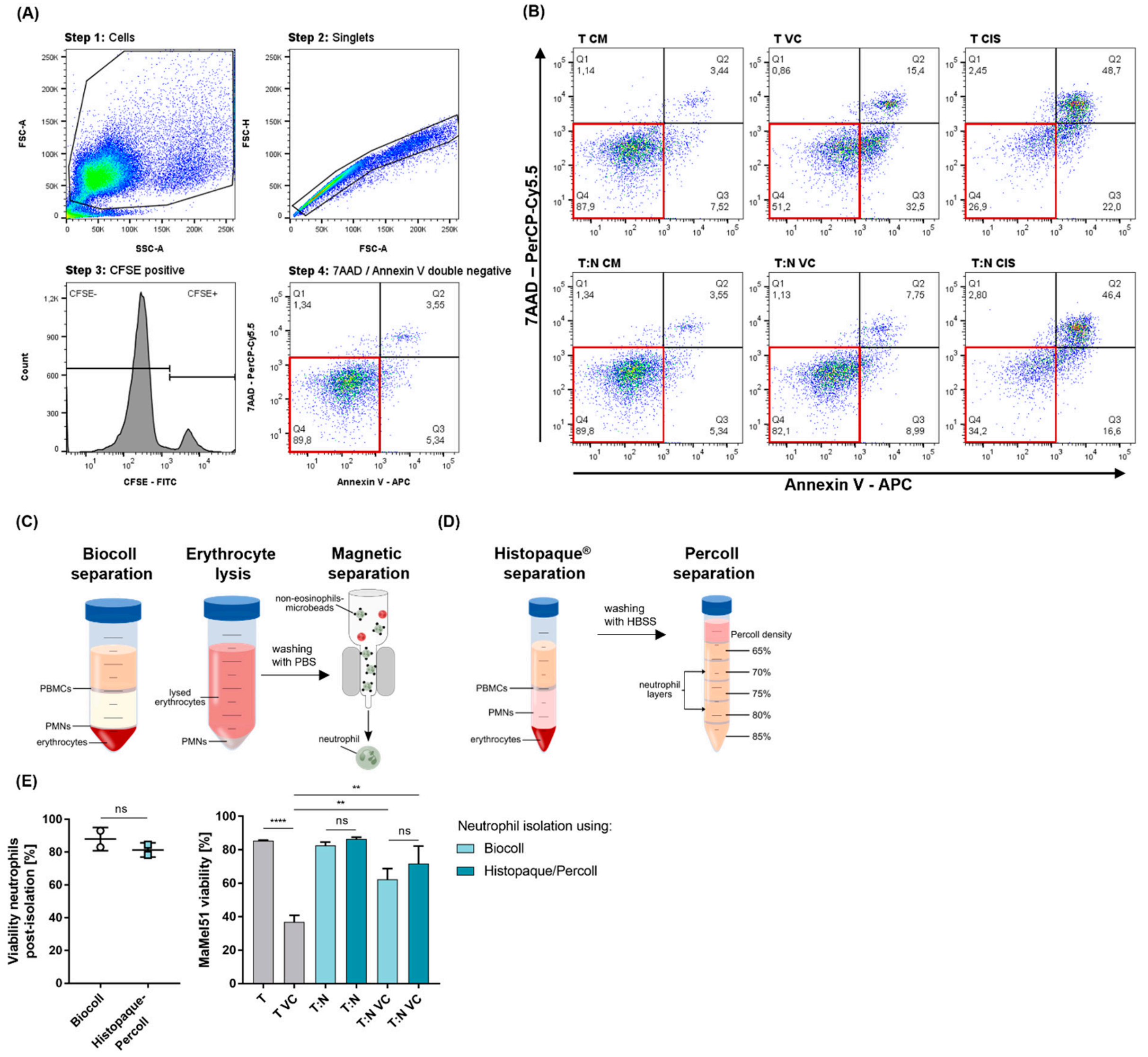

Figure S3. Gating strategies for in vitro viability assays and comparison of two neutrophil isolation techniques. (**A**) Representative gating strategy for viability assessment of MaMel51 cells in cocultures with neutrophils in medium. Cells were identified gating for area of side (SSC-A) and forward scatter (FSC-A) (Step 1), followed by removing doublets gating for height and area of FSC (Step 2). Melanoma cells were identified as CFSE-positive (Step 3) and viable melanoma cells were defined as $7AAD^-$ Annexin V^- as displayed in quadrant 4 (Q4; red box) (Step 4). (**B**) Representative gating strategy for viability assessment of MaMel51 cells (T) in nonadherent cocultures in absence or presence of neutrophils (N) in medium (CM), medium containing 1 μM vemurafenib and 100 nM cobimetinib (VC) or 20 μM cisplatin (CIS) after 24 hours. Viable melanoma cells were defined as $7AAD^-$ Annexin V^- as displayed in quadrant 4 (Q4; red box). (**C**), (**D**), (**E**) Comparison of two different neutrophil isolation methods. (**C**) Isolation of neutrophils using a Biocoll gradient centrifugation, followed by the lysis of erythrocytes using an erythrocyte lysis buffer. For separation of eosinophilic and neutrophilic granulocytes, an automatic labelling-based system, autoMACS pro, using a multi-antibody isolation kit from Miltenyi Biotech was used. Neutrophils represent the positive separation fraction. (**D**) In comparison, neutrophils were isolated, using a Histopaque-based separation method followed by washing with HBSS and an additional Percoll multi-gradient separation. (**E**) The viability (left) of freshly isolated neutrophils prepared in (**C**) and (**D**) was determined following post-isolation. Neutrophil viability was similar for both isolation techniques and constantly > 80 %. The function (right) of neutrophils was determined by their ability to protect MaMel51 cells from treatment with 1 μM vemurafenib and 100 nM cobimetinib (VC) under nonadherent cultures for 24 hours. Both Biocoll and Histopaque isolation methods isolated neutrophils with a similar protection capacity shown as viability increase of MaMel51 cells. Mean percentage of neutrophil viability (left) and MaMel51 viability (right) ±/+ standard deviation (SD) is shown for two independent experiments. ns, $p > 0.05$, ** $p \leq 0.01$ and **** $p \leq 0.0001$.

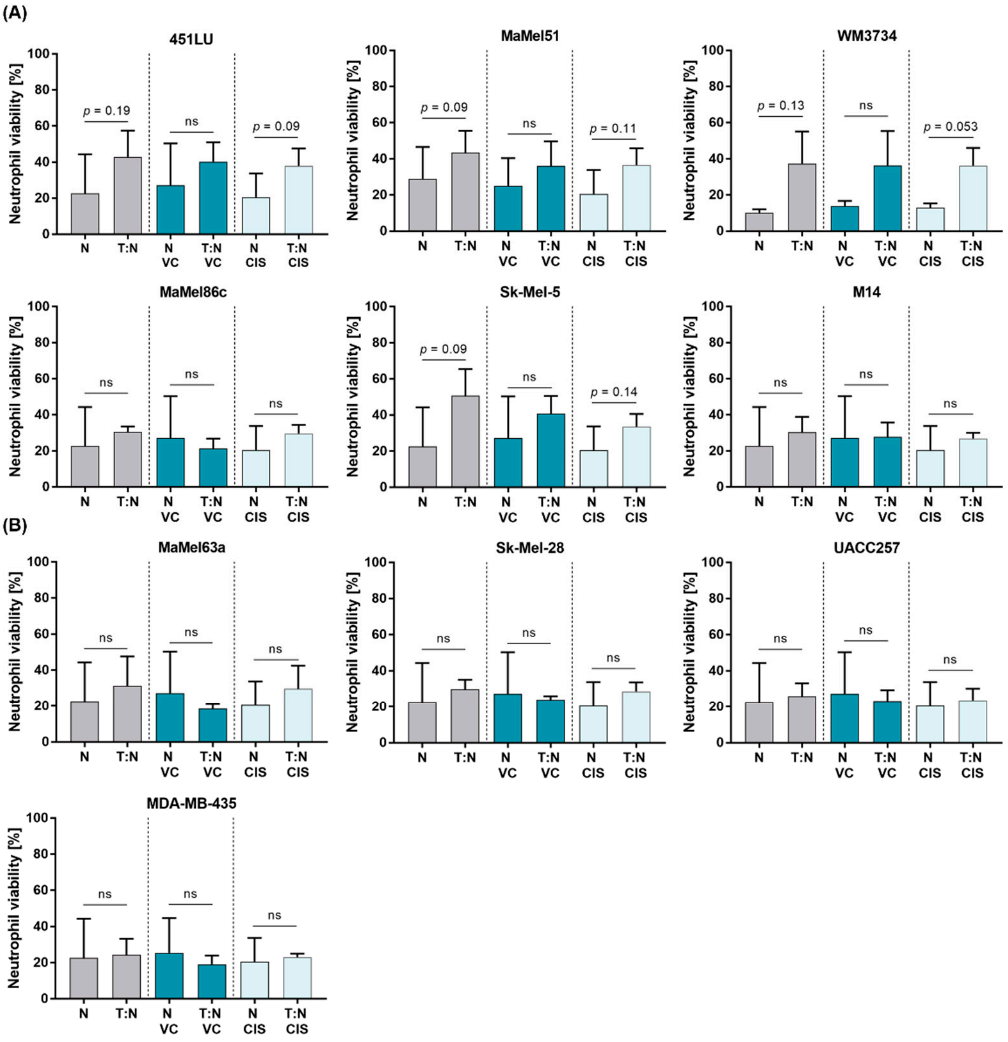

Figure S4. Neutrophil viability was higher in cocultures with melanoma cells showing protectability in in vitro viability assays. (**A**), (**B**) Viability of neutrophils (N) cocultured with or without melanoma cells (T) under nonadherent culture conditions in complete medium, 1 µM vemurafenib and 100 nM cobimetinib (VC) or with 20 µM cisplatin (CIS) for 24 hours. (**A**) Cocultures of neutrophils with protectable cell lines including 451LU, MaMel51, WM3734, MaMel86c, Sk-Mel-5 and M14 demonstrated increased neutrophil viability for conditions with medium and with cisplatin compared to respective control (neutrophils only), which did not reach statistical significance. (**B**) For cultures with unprotectable cell lines including MaMel63a, Sk-Mel-28, UACC257 and MDA-MB-435, such a trend was not observed. Mean viability of neutrophils + SD is shown for three to seven independent experiments. ns, $p > 0.05$.

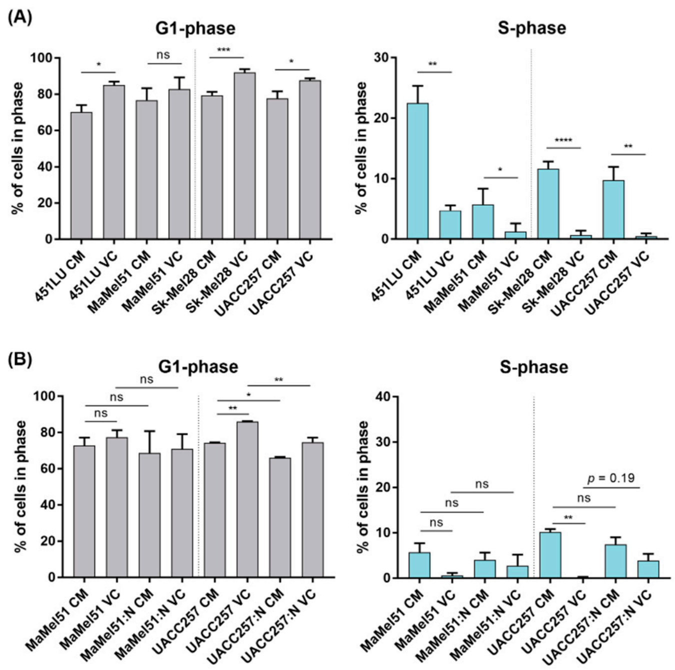

Figure S5. Cell cycle of BRAF-mutated melanoma cell lines is affected by MAPK inhibition and coculture with neutrophils. (**A**) Treatment of two sensitive (451LU and MaMel51) and two low-sensitive (Sk-Mel28 and UACC257) cell lines with 1 µM vemurafenib and 100 nM cobimetinib (VC) under nonadherent culture conditions for 24 hours, subsequent staining with EdU/Hoechst and measurement via flow cytometry. VC treatment induced G1-arrest and S-phase reduction in tested cell lines. The percentage of melanoma cells in each cell cycle phase is shown for two to four independent experiments. (**B**) Coculture with neutrophils induced a reduction in the G1-phase in UACC257 cells compared to the respective control cells without neutrophils. The S-phase was by trend ($p = 0.19$) increased in VC treated UACC257 cells when cocultured with neutrophils compared to the control cells without neutrophils. No significant effect of treatment or culture with neutrophils was observed on the cell cycle of MaMel51 cells. The percentage of melanoma cells + SD in each cell cycle phase is shown for two independent experiments. ns, $p > 0.05$, * $p \leq 0.05$, ** $p \leq 0.01$, *** $p \leq 0.001$ and **** $p \leq 0.0001$.

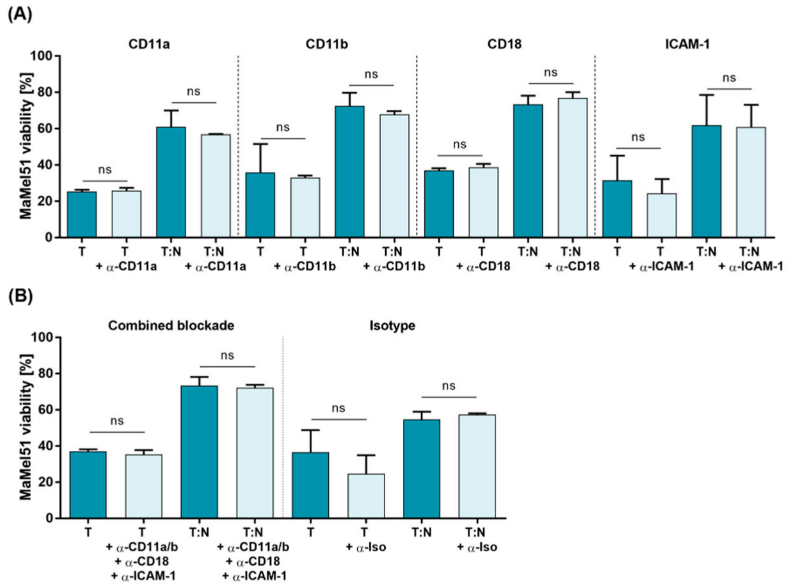

Figure S6. Blocking antibodies did not prevent protection in vitro. Using (**A**) anti-CD11a, anti-CD11b, anti-CD18, anti-ICAM-1 or (**B**) the combination of all antibodies in MaMel51-neutrophil cocultures treated with 1 μM vemurafenib and 100 nM cobimetinib for 24 hours. A respective isotype antibody (α-Iso; anti-IgG_1) was used as control. None of the blocking antibodies prevented neutrophil-induced protection of VC-treated MaMel51 cells. Mean percentage of MaMel51 cell viability + SD is shown for two to three independent experiments. ns, $p > 0.05$.
